# High‐fat diet attenuates estradiol's efficacy on cognitive function and hippocampal synaptic plasticity in middle‐aged ovariectomized mice

**DOI:** 10.14814/phy2.70867

**Published:** 2026-04-10

**Authors:** V. Felintro, L. D. Desmoulins, C. M. Dugas, I. P. dos Santos, S. N. Offor, H. J. Taylor, J. Zha, A. Zsombok, L. A. Schrader

**Affiliations:** ^1^ Department of Cell and Molecular Biology Tulane University New Orleans Louisiana USA; ^2^ Department of Physiology Tulane University New Orleans Louisiana USA; ^3^ Tulane Brain Institute Tulane University New Orleans Louisiana USA

**Keywords:** estradiol, high‐fat diet, hippocampus, synaptic plasticity, spatial memory

## Abstract

Loss of circulating ovarian hormones during menopause is associated with increased risk for cognitive decline in women. Although estradiol has been shown to enhance memory and synaptic function in preclinical studies, clinical studies of hormone therapy in menopausal women are less clear, and its efficacy may be diminished in the context of metabolic dysfunction. This study investigated whether high‐fat diet‐induced obesity impairs the beneficial effects of estradiol on hippocampus‐dependent memory and synaptic plasticity in middle‐aged, ovariectomized female mice. Female C57BL/6J mice were fed a low‐fat or high‐fat diet starting at 9–10 months of age and underwent ovariectomy at approximately 12 months followed by treatment with 17β‐estradiol or vehicle. Spatial memory was assessed using the novel object location task. Long‐term potentiation was measured in acute hippocampus slices via field recordings at the Schaffer collateral‐CA1 synapses. Estradiol treatment significantly improved spatial memory, but the effect of estradiol was attenuated by approximately 36% in high‐fat diet‐fed mice. Estradiol also significantly enhanced long‐term potentiation in slices from low‐fat diet‐fed mice but had no effect in high‐fat diet‐fed mice. These findings suggest that high‐fat diet may interfere with the neuroprotective mechanisms of estrogen within the hippocampus and highlight the importance of metabolic health status in determining the efficacy of hormone therapy during aging.

## 1 INTRODUCTION

Menopause, marked by a decline in ovarian hormone levels, represents a critical transition in female aging. Postmenopausal women face a substantially increased risk of neurodegenerative diseases and cognitive impairments compared with pre‐menopausal women (Hara et al., 2015; McCarrey & Resnick, 2015; Weber et al., 2012). Preclinical studies demonstrate that exogenous estradiol effectively protects against neurodegenerative conditions (Amtul et al., 2010), and can enhance cognitive function (Baumgartner et al., 2022; Boulware et al., 2013; Gresack & Frick, 2006; Kim et al., 2016; Kim & Frick, 2017; Li et al., 2004; Luine & Frankfurt, 2020). These findings suggest that hormone therapy may offer cognitive benefits in postmenopausal women. However, clinical outcomes of hormone therapy vary considerably (Hara et al., 2015; Sullivan Mitchell & Fugate Woods, 2001), with reduced efficacy in women with pre‐existing metabolic disorders, such as type 2 diabetes mellitus (Espeland et al., 2015). These pre‐existing conditions may explain why hormone therapy is ineffective to cognition in some cases.

Notably, obesity and metabolic disorders are well‐established risk factors for cognitive decline, especially in women (Espeland et al., 2015; Sundermann et al., 2021; Yaffe et al., 2004). Evidence suggests that obesity causes insulin resistance and impairs hippocampus‐dependent functions, such as spatial memory by impairing synaptic plasticity (Sanz‐Martos et al., 2024; Underwood & Thompson, 2016). These findings raise the possibility that metabolic dysfunction may interfere with the mechanisms and neural pathways through which estrogens modulate hippocampal activity and cognitive function.

Estradiol is also effective in supporting functional and structural synaptic properties in the hippocampus. Estradiol enhances glutamatergic transmission as well as long‐term potentiation in hippocampal CA1 neurons (Spencer‐Segal et al., 2012; Stelly et al., 2012; Warren et al., 1995) and increases the density of dendritic spines and spine synapses on CA1 pyramidal cells (Woolley et al., 1997; Woolley & McEwen, 1994). These actions are believed to be mediated by hippocampal estrogen receptors which facilitate the activation of intracellular signaling pathways required for maintaining cognitive function during menopause (Spencer‐Segal et al., 2012; Witty et al., 2012).

Given the influence of both obesity and estradiol on neural circuits underlying learning and memory, elucidating the neural substrates and mechanisms by which estrogen modulates cognitive function in healthy and unhealthy conditions is essential for developing effective treatments to prevent cognitive decline in postmenopausal women. To address these gaps, we hypothesized that obesity induced by a high‐fat diet disrupts estradiol's positive effects on hippocampus‐dependent cognition and long‐term potentiation within the hippocampus.

## 2 MATERIALS AND METHODS

### 2.1 Animals

All procedures were performed in accordance with the Tulane University Institutional Animal Care and Use Committee's regulations and are consistent with National Institutes of Health Guidelines for the Care and Use of Laboratory Animals.

Female C57BL6/J mice were obtained from Jackson lab (RRID:IMSR JAX:000664) at 10 weeks of age and housed two per cage in a room maintained at 20°C ± 1°C with a 12/12 h light/dark cycle and ad libitum access to food and water. Mice were maintained on control chow diet for 1 week (Lab Diet, PicoLab Rodent Diet 5053, 13% kcal from fat). One week after arrival, all mice were assigned to a phytoestrogen‐free diet (low‐fat diet, LFD). At approximately 9.5 months of age, mice were randomly assigned to experimental groups and maintained on either the phytoestrogen‐free low‐fat diet (LFD; Bio‐Serv F4031; 16% kcal from fat; 3.93 kcal/g) or a high‐fat diet (HFD; Bio‐Serv F3282; 60% kcal from fat; 5.49 kcal/g) chow.

### 2.2 Ovariectomy and hormone treatment

Approximately 12 weeks after diet initiation, all mice were ovariectomized bilaterally. Briefly, anesthesia was initially induced with 5% isoflurane in an induction chamber and maintained with 1.0%–2.0% isoflurane, adjusted as necessary for appropriate depth of anesthesia. To prevent corneal desiccation, a thin layer of antibacterial ophthalmic ointment (Akorn, Illinois, USA) was applied to the eyes. Mice were subcutaneously administered Meloxicam (5 mg/kg) preoperatively to minimize discomfort and inflammation. The incision areas were cleaned with betadine and alcohol solution, and a small incision was made at T12‐L2. The ovaries and surrounding adipose tissue were removed and a hemostat was placed at the boundary between the oviducts and uterus, and a nonabsorbable suture placed just below the hemostat. The ovaries were dissected out and the uterus was returned to the abdomen. All incisions were closed in two layers with interrupted polydioxanone absorbable sutures. To determine the effect of E2 treatment, silastic capsules (1.47 mm ID, 1.96 mm OD; Dow Corning #508‐006) were prepared containing either 2.5% 17 β‐Estradiol (E2, Sigma‐Aldrich E8875) dissolved in cholesterol (Sigma Aldrich, 228111) or 100% cholesterol alone (vehicle, VEH). Capsules were subcutaneously implanted at the base of the neck and remained in place for 40 days, resulting in the following four experimental groups: (1) LFD‐VEH, (2) LFD‐E2, (3) HFD‐VEH and (4) HFD‐E2. This level of estradiol has been shown to maintain circulating levels of E2 in the physiological range (Molinas et al., 2025). All mice were monitored following surgery and after recovery were pair‐housed with a mouse of the same diet and treatment. The capsule area was massaged once per week to prevent scar tissue formation. Throughout the study, the mice continued their respective diets.

### 2.3 Novel object location task

Approximately 40 days after ovariectomy surgery, spatial memory was tested using the Novel Object Location task as previously described (Vogel‐Ciernia & Wood, 2014). Briefly, middle‐aged female mice were habituated to an open‐field arena (50 × 50 × 50 cm) over two consecutive days (habituation sessions), with 5 min of free exploration per day. On the third day (training session), two identical objects were positioned in diagonally opposite corners, and mice were allowed unrestricted exploration for 5 min. Twenty‐4 h later (test session), spatial memory was assessed by relocating one of the objects to a novel location within the arena. Mice were then reintroduced for an additional 5‐min session, during which exploration times for both novel and familiar object locations were recorded and subsequently analyzed by I.P. dos Santos and H.J. Taylor using the Boris analysis software. Head‐directed object interaction within 3 cm of the object was counted as object observation.

The discrimination index, calculated as the ratio of exploration time spent with the object in the novel location relative to the total exploration time of both objects, was used as the measure of spatial memory retention. To minimize olfactory cues between trials, the arena was thoroughly cleaned with water after each session and disinfected with 70% ethanol at the end of each testing day. All behavioral experiments were conducted during the animals' light phase to ensure circadian consistency. Behavioral tracking and data acquisition were performed using EthoVision XT software (Noldus Information Technology).

Mice exhibiting unequal exploration (<30% of total time spent exploring one object) during the training phase, or less than 3 s of exploration time for either object during training or testing phases, were excluded from analysis (*n* = 2 LFD; *n* = 2 HFD; *n* = 3 LFD + E2; *n* = 2 HFD + E2). Additionally, animals showing a preference of less than 30% exploration of the relocated object during the test session were excluded, as this behavior may suggest neophobia rather than impaired spatial memory (*n* = 1 HFD).

### 2.4 Brain slice preparation

Three‐four weeks after behavior testing and when the mice were 14–16 months of age, mice were anesthetized with isoflurane and immediately decapitated. Brains were rapidly extracted and immersed in iced‐cold N‐methyl‐D‐glucamine (NMDG)‐based slicing solution (in mM: 110 NMDG, 110 HCL; 3 KCl; 1.1 NaH_2_PO_4_; 3 pyruvic acid; 10 ascorbic acid; 25 D‐glucose; 25 NaHCO_3_, 10 MgCl_2_. 0.5 CaCl_2_), saturated with 95% O_2_/5% CO_2_ (vol/vol), pH 7.3–7.4. Horizontal hippocampal slices (400 μm) were prepared using a Series 3000 Plus vibratome in chilled, carbogen‐bubbled NMDG solution to maintain tissue viability. The freshly isolated hippocampal slices were immediately transferred to a holding chamber with oxygenated NMDG solution for 15 min. Next, slices were placed in oxygenated artificial cerebrospinal fluid (aCSF; in mM: 124 NaCl, 2.5 KCl, 1.06 NaH_2_PO_4_, 25 NaHCO_3_, 14.5 glucose, 2 MgCl_2_, 2 CaCl_2_, pH 7.3–7.4) and incubated at room temperature for at least 1 h prior to experimental use.

### 2.5 Electrophysiology recordings

A dorsal hippocampal slice was transferred to a submerged recording chamber and bathed with oxygenated aCSF at a controlled flow rate of 1.5~2 mL/min at room temperature. Synaptic responses in the CA1 region were elicited by delivering electrical stimuli via a bipolar tungsten electrode strategically placed in the CA3 Schaffer collateral fibers of the hippocampus. A recording pipette filled with aCSF was positioned in the *stratum radiatum* of CA1 to record field excitatory postsynaptic potentials (fEPSPs). Input–output curves were established by increasing the stimulation intensity in 0.1 mA increments until a maximal synaptic response. Baseline synaptic transmission was recorded at 0.1 Hz with stimulus intensity set to 50% of the maximal fEPSP slope, stabilized over at least 10 min. Long term potentiation was induced using a theta‐burst stimulation protocol, consisting of three trains separated by 20 s, each comprising 10 bursts at 5 Hz, with each burst containing 4 pulses delivered at 100 Hz, applied at the baseline stimulus intensity. Post‐TBS, fEPSPs were recorded at 0.1 Hz for at least 60 min. The fEPSP slope was calculated as the linear fit of the 10%–90% rising phase of the peak amplitude of the fEPSP. Six consecutive fEPSPs recorded within each minute were averaged to generate a single slope value per minute. Synaptic strength was quantified by normalizing the initial fEPSP slope to the mean baseline responses of each animal. Data were acquired using a MultiClamp 700B amplifier (Molecular Devices) and analyzed with Clampfit 10.7 software. A subset of animals was used for electrophysiology recordings and 1 recording was made per slice (5 LFD‐VEH, 7 slices; 5 HFD‐VEH, 7 slices; 5 LFD‐E2, 7 slices; and 6 HFD‐E2, 11 slices). The data that support the findings of this study are available from the corresponding author upon reasonable request.

### 2.6 Statistical analysis

Unless otherwise noted, all values are expressed as means ± SD. Data normality was evaluated using the Shapiro–Wilk test, and outliers were identified and excluded using Grubbs' test; normality was satisfied for all data. Group comparisons were made using two‐way analysis of variance (ANOVA), followed by simple effect tests. Simple effects tests were planned a priori due to expected group differences in the low‐fat diet groups. In the novel object location test, since the discrimination index depends on exploration of both objects, one‐sample *t*‐tests were run on each group to measure statistical difference from chance, where equal exploration of each object is chance (0.5) (Gresack & Frick, 2003; Gresack & Frick, 2006). The Geisser–Greenhouse correction was used when repeated measures analyses violated the sphericity assumption. Nested model analysis was used when multiple data points were taken from the same animal (electrophysiology). Statistical analyses were performed using SPSS (version 29) and GraphPad Prism software (version 10.3, San Diego, CA, USA). A *p*‐value of ≤0.05 was considered statistically significant. Detailed descriptions of the specific statistical tests are provided in each legend.

## 3 RESULTS

### 3.1 Effects of estradiol and diet on uterine and body weights

**FIGURE 1:**
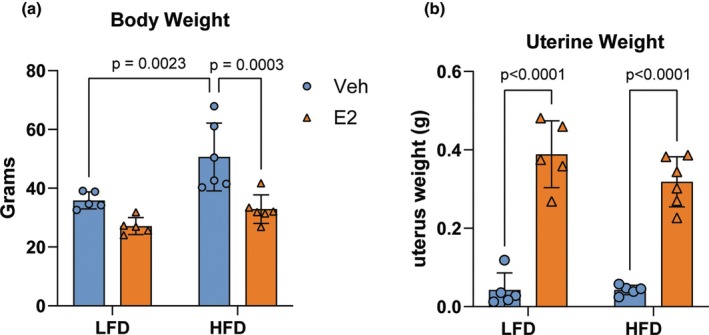
The impact of estradiol and diet on body and uterine weight. (a) Body weight and (b) uterine weight. Mice fed with high‐fat diet (HFD) showed significantly increased body weight compared with low‐fat diet‐fed (LFD) animals. Estradiol treatment significantly reduced that weight gain in high fat diet‐fed mice. (b) Mice treated with estradiol (E2) exhibited significantly heavier uterine weights compared with vehicle‐treated (VEH) mice. Data are presented as mean ± SD (*n* = 5–6 per group).

Given the established role of estradiol in regulating both metabolic and reproductive functions (Acharya et al., 2023; Gloy et al., 2011; Ziomkiewicz et al., 2008), we first evaluated the systemic effects and efficacy of estradiol treatment on uterine and body weight (See Figure 1). In the current study, the animals used for long‐term potentiation studies were assessed for body weight and uterine weights. Regarding body weight, two‐way ANOVA revealed significant main effects of estradiol treatment (*F*(1, 18) = 20.28, *p* = 0.0003, *η*_p_^2^ = 0.53) and diet (*F*(1, 18) = 12.19, *p* = 0.0026, *η*_p_^2^ = 0.40), with no significant interaction between factors (*F*(1, 18) = 2.35, *p* = 0.1430, *η*_p_^2^ = 0.11) (Figure 1a). Simple effects tests showed that estradiol‐treated mice in the high‐fat‐fed groups weighed significantly less compared with their respective vehicle‐treated controls (HFD‐E2 VS HFD‐VEH *F*(1, 18) = 20.03, *p* < 0.0003, *η*_p_^2 =^ 0.53); there was a nonsignificant trend of lower magnitude in the low‐fat diet‐fed group (LFD‐E2 vs. LFD‐VEH, *F*(1, 18) = 4.05, *p* = 0.06, *η*_p_^2^ = 0.183). These findings demonstrate that estradiol mitigates weight gain under high‐fat diet conditions.

As expected, estradiol increased uterine weights (*F*(1, 17) = 148.8, *p* < 0.0001, *η*_p_^2^ = 0.90; the uterus from one animal was not measured; Figure 1b), with no significant effect of diet (*F*(1, 17) = 1.87, *p* = 0.19, *η*_p_^2^ = 0.10) or interaction between diet and treatment (*F*(1, 17) = 1.90, *p* = 0.187, *η*_p_^2^ = 0.094). Simple effects tests showed that uterine weights were significantly increased in estradiol‐treated mice, both in the low‐ and high‐fat‐fed groups (LFD‐E2 vs. LFD‐VEH, *F*(1, 17) = 88.32, *p* < 0.00001, *η*_p_^2^ = 0.839; HFD‐E2 vs. HFD‐VEH F(1, 17) = 61.234, *p* < 0.00001, *η*_p_^2 =^ 0.783). These findings demonstrate that estradiol treatment effectively increases uterine weight in both low‐ and high‐fat diet conditions, confirming the biological activity of the hormone and that the subcutaneous capsule implantation successfully released estradiol.

### 3.2 Estradiol treatment enhances novel object location memory in middle‐aged ovariectomized mice

**FIGURE 2:**
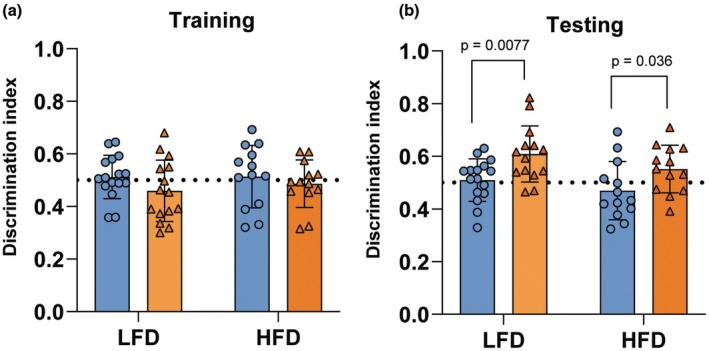
Estradiol enhances memory in low‐fat diet with an attenuated effect in high‐fat diet‐fed mice. (a) Graph showing the discrimination index during training and (b) test session. Error bars are presented as mean ± SD (*n* = 13–16 per group). LFD‐E2 is the only group that was significantly different than chance. Each data point represents an individual mouse.

Previous studies have shown that estradiol treatment enhances spatial memory in rodents (Baumgartner et al., 2022; Boulware et al., 2013; Gresack & Frick, 2006; Kim et al., 2016; Kim & Frick, 2017; Li et al., 2004), and the novel object location task involves dorsal hippocampus function (Cohen et al., 2013). Most studies focus on estradiol treatment in healthy animals; therefore, we aimed to determine whether estradiol treatment could alter memory in both healthy and diet‐induced obese conditions. To investigate the effects of estradiol on spatial memory, we performed the novel object location test (Figure 2). During the training phase, the discrimination indices for each group were not different from chance (0.5, denoted by the horizontal dotted line in Figure 2; *p* > 0.05), and there were no significant differences in the discrimination index between the groups, indicating similar levels of object exploration across all conditions during training (two‐way ANOVA; all *p* > 0.05; Figure 2a). This confirms that baseline object exploratory behavior was comparable among groups, allowing a valid comparison of memory performance during the test phase. In the test phase, only the low‐fat diet‐fed estradiol‐treated group showed significant difference from chance (0.5) (*t*(13) = 3.8; *p* = 0.002), while the high‐fat diet estradiol‐treated group showed a trend (*t*(12) = 1.84; *p* = 0.090). A significant main effect of estradiol treatment was detected on the two‐way ANOVA (*F*(1, 52) = 12.03, *p* = 0.001, *η*_p_^2^ = 0.18; Figure 2b), demonstrating that estradiol treatment significantly improved spatial memory performance on the novel object location memory task. No significant interactions or differences were observed between the HFD and LFD groups within the same treatment (Interaction: *F*(1, 52) = 0.097, *p* = 0.76, *η*_p_^2^ = 0.002; Diet: *F*(1, 52) = 3.49, *p* = 0.07, *η*_p_^2^ = 0.063). Simple effects tests showed that estradiol‐treated mice, both in the low‐ and high‐fat‐fed groups displayed significantly higher discrimination indices compared with their respective vehicle‐treated controls LFD‐E2 vs. LFD‐VEH, (*F*(1, 52) = 7.68, *p* = 0.0077, *η*_p_^2^ = 0.129; HFD‐E2 vs. HFD‐VEH *F*(1, 52) = 4.66, *p* = 0.036, *η*_p_^2^ = 0.082). Thus, although estradiol supported memory in both groups, the variance was attenuated by approximately 36% under high‐fat diet compared with low‐fat diet.

### 3.3 Impact of estradiol on synaptic plasticity: Differential effects based on dietary conditions

**FIGURE 3:**
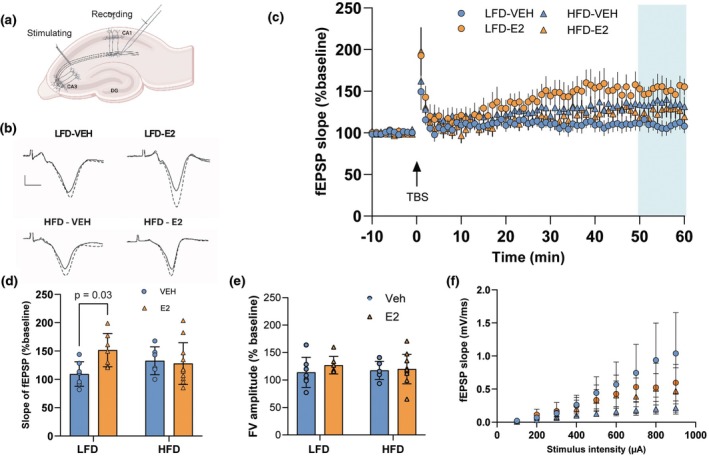
Estradiol increases long‐term potentiation in low‐fat but not in high‐fat diet‐fed mice. (a) Schematic of the hippocampal slice preparation with experimental settings for field excitatory postsynaptic potential (fEPSP) recordings in the CA1 region. (b) Example of fEPSP traces showing typical responses. Scale bar = 10 μV/10 ms. (c) Time course of changes in fEPSP slope following theta‐burst stimulation, data presented as mean ± SEM for clarity. (d) Quantification of fEPSP slope 50–60 min post‐TBS. (e) Summary bar graph of the percent change in fiber volley amplitude 50–60 min post‐TBS. (f) Input–output curves representing fEPSP slope across increasing stimulation intensities. Data are presented as mean ± SEM for clarity; (*n* = 6–11 per group). Each data point represents an individual slice.

Long‐term potentiation of synapses is considered a cellular correlate for learning and memory (Abraham et al., 2019). To investigate a neural substrate for the estradiol‐enhanced memory, we investigated the role of estradiol in long‐term potentiation in hippocampus slices from the low‐ and high‐fat diet‐fed mice. We conducted extracellular field recordings from the Schaffer collateral‐CA1 synapses in dorsal hippocampal slices of middle‐aged ovariectomized mice, focusing on the effects of estradiol and diet (HFD vs. LFD) on synaptic plasticity (Figure 3). We used theta burst stimulation, described in methods, to induce LTP. This stimulation paradigm induced strong LTP in young ovariectomized animals (Maroteaux et al., 2024). Our data showed that estradiol significantly enhanced long‐term potentiation in the low‐fat diet group, but not in high‐fat diet‐fed animals (Figure 3c,d). The measures from the last 10 min of recordings (50–60 min after TBS) were averaged and the numbers from each group compared. Because we had 1–3 slices for each animal, we conducted a two‐way nested model ANOVA. This analysis comparing the last 10 min of long‐term potentiation recordings (Figure 3c) revealed a significant interaction between diet and estradiol treatment (*F*(1, 21.06) = 5.46, *p* = 0.03, *η*_p_^2^ = 0.206), indicating that the effects of estradiol on long‐term potentiation depend on diet. Simple effects tests showed that estradiol‐treated mice in the low‐fat diet fed groups displayed significantly higher magnitude of LTP compared with their respective vehicle‐treated controls but no effects in the high‐fat diet‐fed animals LFD‐E2 vs. LFD‐VEH, (*F*(1, 21.06) = 5.51, *p* = 0.03, *η*_p_^2^ = 0.207; HFD‐E2 vs. HFD‐VEH *F*(1, 21.06) = 0.87, *p* = 0.36 *η*_p_^2^ = 0.039). Thus, whereas estradiol accounted for less than 4% of the variance in synaptic plasticity in animals fed high‐fat diet, it accounted for over 20% of the variance in animals fed low‐fat diet. This indicates that estradiol promotes synaptic plasticity in the hippocampus in a metabolically healthy context but fails to produce the same effect in obese mice.

The amplitude of the fiber volley, which reflects presynaptic fiber activation, was analyzed to determine whether any presynaptic mechanisms might contribute to the observed effects. The two‐way nested model ANOVA showed no significant interaction between treatment and diet (*F*(1, 20.12) = 0.35, *p* = 0.56; Figure 3e) and no main effects of diet (*F*(1, 20.1) = 0.54, *p* = 0.47) or estradiol treatment (*F*(1, 20.12) = 0.12, *p* = 0.74), indicating that neither estradiol nor diet had a significant impact on presynaptic fiber activation.

An input–output curve (Figure 3f) was performed to assess synaptic efficacy. A repeated‐measures three‐way ANOVA indicated a significant main effect of stimulus intensity (*F*(1.09, 28.23) = 6.43, *p* < 0.02) but no significant effect of diet (*F*(1, 26) = 1.15, *p* = 0.293; or E2 *F*(1, 26) = 0.019, *p* = 0.89 or diet × hormone interaction *F*(1, 26) = 0.355, *p* = 0.556). Overall, these results suggest that neither estradiol nor diet affects baseline synaptic activity prior to long‐term potentiation induction.

## 4 DISCUSSION

The current study investigated the impact of estradiol and high‐fat diet on hippocampus function in ovariectomized middle‐aged female mice. Our results provide evidence that estradiol treatment enhances spatial memory in middle‐aged, ovariectomized female mice, but the effectiveness of estradiol is attenuated by approximately 36% in high‐fat diet conditions. Interestingly, estradiol did reduce the weight gain effects observed in high‐fat diet‐fed mice. Together, these results suggest that the brain and the hippocampus specifically, are possible loci for inconsistent cognitive results of hormone treatment observed in metabolic disorders. These findings have important implications for understanding how metabolic status modulates the neuroprotective effects of estrogen, particularly in the context of cognitive aging and postmenopausal hormone therapy.

In our study, estradiol treatment resulted in markedly reduced body weight in high‐fat diet‐fed mice. Consistent with our results on weight in rodents, ovariectomy is associated with decreased physical activity and increased food intake, while estradiol treatment in ovariectomized animals prevents weight gain, supporting a protective role of estradiol against diet‐induced obesity (Acharya et al., 2023; Bless et al., 2014; Stubbins et al., 2012). As expected, uterine weight was significantly increased in estradiol‐treated groups, confirming effective estradiol treatment and activation of peripheral estrogen receptors.

High‐fat diet has been linked to memory impairments and increased risk of neurodegeneration in male animals (Anstey et al., 2011; Beydoun et al., 2008; Miller & Spencer, 2014). Previous studies showed that male rodents exposed to a high‐fat diet showed impaired hippocampus‐dependent memory (Arnold et al., 2014; Stranahan et al., 2008; Wu et al., 2003). On the other hand, intact female mice are resistant to the effects of high‐fat diet regarding weight gain and metabolic changes, as well as hippocampus function, including memory formation and synaptic plasticity (Hwang et al., 2010; Sanz‐Martos et al., 2024), suggesting that normally circulating ovarian hormones in females may contribute to the protective impacts in high‐fat diet‐fed mice.

Consistent with previous research, estradiol significantly improved performance on the novel object location task. However, only the low‐fat diet treated with estradiol group displayed a memory preference significantly above chance, suggesting that estradiol's efficacy is diminished under metabolically compromised conditions. Interestingly, estradiol enhances object recognition memory in young (5 months) and middle‐aged (17 months) but not aged (22 months) ovariectomized mice, suggesting plasticity may be coupled to differential pathways in aged animals (Gresack et al., 2007). Similar mechanisms may be at play in middle‐aged unhealthy animals, as in our case, the high‐fat diet‐fed animals.

Activity‐dependent remodeling of synapses that underlies long‐term potentiation provides a neurobiological basis for memory formation. Therefore, we investigated the effects of estradiol and diet on hippocampal long‐term potentiation. While there was a lack of correlation between the LTP and discrimination index measured in the novel object location, our electrophysiological data support our behavioral results and suggest the hippocampus is one possible locus for estradiol's effects on memory. In this study, we show that estradiol enhances long‐term potentiation in slices from low‐fat diet‐fed mice. In contrast, slices from high‐fat diet‐fed mice failed to show a comparable long‐term potentiation enhancement following estradiol treatment, despite confirmation of systemic estradiol activity through increased uterine weight and body weight attenuation. Importantly, we found that neither estradiol treatment nor high‐fat diet significantly altered presynaptic fiber activation or input–output curves, indicating that the observed effects on long‐term potentiation were likely due to postsynaptic mechanisms; however, more investigation is necessary. This is consistent with literature suggesting that estradiol's enhancement of plasticity involves upregulation of postsynaptic glutamate receptor activity, dendritic spine formation, and synapse remodeling (Kramar et al., 2009; Liu et al., 2008; Woolley & McEwen, 1994).

The mechanisms of the lack of effect of estradiol in high‐fat diet‐fed mice require further investigation. Various plasticity mechanisms may be disrupted in the context of obesity, potentially due to increased oxidative stress and neuroinflammation (de Paula et al., 2021; Evans et al., 2024), dysregulated protein degradation pathways (McFadden et al., 2020), or regulation of plasticity‐related miRNAs (Spinelli et al., 2024). Furthermore, these findings suggest that the intracellular signaling mechanisms necessary for estradiol's actions on synaptic plasticity, such as PI3K/Akt, MAPK, and NMDA receptor‐dependent pathways (Boulware et al., 2013; Fan et al., 2010; Kim et al., 2016; McFadden et al., 2020; Spencer‐Segal et al., 2012), may be compromised by chronic metabolic stress, as reported in previous studies.

Finally, neuroestrogens locally produced in the hippocampus by the enzyme aromatase are necessary for memory consolidation (Tuscher et al., 2016) and long‐term potentiation in female mice (Maroteaux et al., 2024; Vierk et al., 2012; Wang et al., 2018). While high‐fat diet increases aromatase gene expression (CYP19A1) in other tissues (Chen et al., 2012; Polari et al., 2015), the influence of high‐fat diet on aromatase activity and/or expression in the hippocampus is not known, but may also be a locus for disruption of plasticity‐related mechanisms.

Some limitations should be acknowledged in this study. First, analyses were restricted to the hippocampus, leaving open the possibility that estradiol may exert differential effects in other brain regions involved in memory and cognitive integration. Indeed, investigation in the hypothalamus showed that the firing of liver‐related paraventricular nucleus neurons was significantly reduced compared with vehicle‐treated HFD mice, whereas in LFD mice, estradiol treatment did not alter the activity of liver‐related PVN neurons (Molinas et al., 2025). Second, findings from middle‐aged female mice may not generalize to younger/older age groups, given known age‐dependent hormonal sensitivities. Finally, spatial memory was assessed using a single behavioral task, which may not fully capture the complexity of estrogen–diet interactions across different memory systems. Indeed, we cannot rule out the possibility that estradiol enhances, or HFD reduces, novelty preference (Swiercz et al., 2025). It's important to note that the hippocampus, prefrontal cortex, and striatum are important for novel object attention (Wulaer et al., 2020). Future studies should investigate whether brain‐region and specific mechanisms, such as cortical compensation, could provide insight into potential adaptive responses that mitigate hippocampal dysfunction.

In summary, estradiol effectively improved spatial memory and long‐term potentiation in middle‐aged low‐fat diet‐fed female mice, but these benefits were diminished in high‐fat diet‐fed animals. Although estradiol reduced the body weight in high‐fat diet‐fed mice, it failed to restore hippocampal function, suggesting a specific impairment at the neuronal level. These findings highlight the critical role of metabolic context in modulating estradiol efficacy both peripherally and centrally and may help explain the limited cognitive benefits of hormone therapy observed in clinical populations with obesity or metabolic syndrome.

## AUTHOR CONTRIBUTIONS

**V. Felintro:** Data curation; formal analysis; investigation; resources. **L. D. Desmoulins:** Conceptualization; data curation; formal analysis; investigation; methodology. **C. M. Dugas:** Data curation; formal analysis; investigation; methodology; resources. **I. P. dos Santos:** Data curation; formal analysis; investigation; methodology. **S. N. Offor:** Data curation; formal analysis; investigation. **H. J. Taylor:** Data curation; formal analysis; investigation. **J. Zha:** Conceptualization; data curation; formal analysis; funding acquisition; investigation; methodology. **A. Zsombok:** Conceptualization; formal analysis; funding acquisition; investigation; methodology. **L. A. Schrader:** Conceptualization; data curation; formal analysis; funding acquisition; investigation; methodology; resources; supervision.

## FUNDING INFORMATION

This study was supported by the National Institute of Health (NIH) through the National Institute on Aging P01AG071746–Estrogens, Cardiometabolic Health, and Female Cognitive Aging.

## CONFLICT OF INTEREST STATEMENT

Authors report no conflict of interest.

## ETHICS STATEMENT

All procedures were approved by the Institutional Animal Care and Use Committee (IACUC) of Tulane University, Louisiana, USA (approval numbers: ID 1490 and 2291), and were conducted in accordance with the National Institutes of Health Guide for the Care and Use of Laboratory Animals.

## Data Availability

The data that support the findings of this study are available from the corresponding author (LAS) upon reasonable request.
